# Caterpillar-induced plant-soil feedback affects resistance in wild and cultivated cabbage

**DOI:** 10.1007/s11104-026-08355-4

**Published:** 2026-02-11

**Authors:** Kris A. de Kreek, Rieta Gols, Johannah M. de Zeeuw, Ilva van Dam, Rob Nijhof, Brigitte S. Noordijk, Marcel Dicke, Karen J. Kloth

**Affiliations:** Laboratory of Entomology, Wageningen University & Research, PO Box 16, 6700 AA Wageningen, the Netherlands

**Keywords:** Plant-soil feedback, *Mamestra brassicae*, *Brassica oleracea*, Intraspecific variation, Jasmonic acid

## Abstract

**Background and aims:**

Aboveground insect herbivory can change the plant rhizosphere and modulate the composition of the soil microbiome. However, it is unclear to what extent these changes in the rhizosphere affect plant resistance to above-ground herbivorous insects, and how these plant-soil feedback (PSF) mechanisms are shaped. Here, we investigated whether herbivore-induced changes in the rhizosphere increase resistance against caterpillars in cabbage, *Brassica oleracea*, and how intraspecific variation of the host plant, herbivory intensity, and soil type affect PSF outcomes.

**Methods:**

PSF experiments with rhizosphere-soil transfer were performed for a wild and cultivated *B. oleracea*, with different densities of the caterpillar *Mamestra brassicae*, and different soil types.

**Results:**

We found that caterpillar-induced soil conditioning affected the performance of *M. brassicae* feeding on the shoot, depending on both intraspecific variation of the host plant and the intensity and duration of herbivory. On wild cabbage, caterpillar-induced PSF positively affected plant resistance to *M. brassicae*, which needed more than two weeks to become detectable. In contrast, in cultivated cabbage, caterpillar-induced PSF had a neutral to negative effect on plant resistance and did not differ between soil types. The observed negative PSF effect was associated with downregulation of genes involved in jasmonic acid biosynthesis and downstream signalling.

**Conclusion:**

Overall, we found that natural variation within one plant species can, depending on intensity and duration of herbivory, result in opposite PSF effects with consequences for jasmonic acid-mediated defences.

**Supplementary Information:**

The online version contains supplementary material available at 10.1007/s11104-026-08355-4.

## Introduction

Plant growth, productivity and reproduction depend strongly on soil properties, such as water and nutrient content, and presence of beneficial or pathogenic macro- and microorganisms. Plants can influence these soil properties and induce changes in the soil that can be preserved as a soil legacy (van der Putten et al. 2013). Soil legacies can have long-lasting effects on current and future vegetation (Heinze and Joshi 2018). This so-called plant-soil feedback (PSF) can be negative when toxic compounds and pathogens accumulate or when nutrients become depleted (Bennett and Klironomos 2019). It can result in positive effects on plant fitness when organic matter, allelopathic chemicals, and beneficial macro- and microorganisms accumulate in the soil. These beneficial organisms can increase bioavailability of nutrients, retain water, enhance plant growth, and enhance resistance to biotic and abiotic stress (Bai et al. 2022; Bennett and Klironomos 2019; Friman et al. 2021c). The sum of all these contributions determine the strength and directions of overall PSF (van der Putten et al. 2013).

The multitude and complexity of soil-borne factors make the outcome of PSF often context dependent. For instance, soil type is a strong determinant of PSF due to distinct chemical and microbial compositions (Bai et al. 2022; Gfeller et al. 2023; Wagner and Mitchell-Olds 2023). In addition, different plant species require nutrients in distinct quantities and qualities and accumulate different beneficial and pathogenic microorganism (Semchenko et al. 2022; van der Putten et al. 2013). This is the reason that plant diversification and crop rotation are used in agricultural practices to prevent pathogen accumulation and depletion of essential nutrients (Mariotte et al. 2018; van der Putten et al. 2013). As the soil ecosystem is not static, soil-borne legacies are also subjected to change. Temporal dynamics in, for example, plant phenology and the soil microbiome therefore, lead to variation in PSF (Hannula et al. 2021; Kardol et al. 2013). Over a time span of multiple plant generations, continuous growth of a plant species and its exposure to biotic stressors can result in a disease suppression in the soil, for example, by promoting growth of a beneficial microbiome (Berendsen et al. 2018; Howard et al. 2020; Raaijmakers and Mazzola 2016). On a shorter time span, PSF can be changed by newly grown vegetation within a few weeks (Steinauer et al. 2023).

Insect herbivory can affect PSF and change insect-plant interactions for plants growing subsequently on that soil (Bezemer et al. 2013; Friman et al. 2021a; Heinen et al. 2022; Yang et al. 2025). It has been shown that biotic-stress-induced PSF effects depend on e.g. the host plant, but also the herbivore type and species (Friman et al. 2021a; Heinen et al. 2022). For example, aboveground feeding by the caterpillar *Mamestra brassicae* on *Jacobaea vulgaris* induces changes in the soil that increase growth of *M. brassicae* caterpillars in the next plant generation. Belowground feeding by the wireworms *Agriotes lineatus* on the same host increases resistance to *M. brassicae* via PSF (Kostenko et al. 2012). Interestingly, the strength of PSF also depends on the intensity of herbivory on receiver plants (Heinze et al. 2019) and may thereby affect larval development and insect reproduction. These and other studies illustrate the complexity of herbivory-induced PSF responses.

Eventually, PSF can affect insect-plant interactions via the modulation of plant defences (Yang et al. 2025). These defences can involve physical structures, such as trichomes and epicuticular wax layers, and chemical defences, such as the production of toxic secondary metabolites, reactive oxygen species and protease inhibitors (Schoonhoven et al. 2005). These defences are generally constitutively expressed, but they can also be upregulated by herbivory (Ali et al. 2024; Schoonhoven et al. 2005), both locally and systemically. Already within minutes signals underlying induced defences may spread within the plant between above- and belowground compartments (Kloth and Dicke 2022). Phytohormones, such as jasmonic acid (JA) and salicylic acid (SA) are important regulators of systemic herbivore-induced defences (Ali et al. 2024; Erb et al. 2012; Soler et al. 2013) and their activity can be affected by PSF (Blundell et al. 2020; Zhu et al. 2018). Intraspecific variation in these defence traits occurs in many plant species, and this variation can be large between cultivated crops and wild relatives (Whitehead et al. 2017). Domestication may have resulted in partial loss of defence traits as these traits were not the ones selected for by breeders (Bernal and Medina 2018; Whitehead et al. 2017). Similarly, there are indications that domestication has also affected PSF by loss of root traits that are relevant for the interaction with the soil and its macro- and microbiome (Carrillo et al. 2019; Mariotte et al. 2018; Martin-Robles et al. 2020).

The soil microbiome plays a large role in PSF, and plants can influence the composition of the microbiome within and around their roots (Semchenko et al. 2022; Steinauer et al. 2023; Yang et al. 2025). For instance, pathogen infection of *Arabidopsis thaliana* by *Hyaloperonospora arabidopsidis* increases the abundance of three bacterial strains in the rhizosphere. These strains enhance disease suppression in later plant generations growing on that soil (Berendsen et al. 2018). These kind of changes in microbiome community may be steered by the plant via root exudation of primary and secondary metabolites. Root exudates can have antimicrobial activities or serve as a substrate for microbial growth. Release of these metabolites into the rhizosphere result in the assembly of a beneficial microbiome with positive PSF effects on the next generation of plants (Bai et al. 2022; Bouwmeester et al. 2025). In line with this, *Spodoptera frugiperda*-caterpillar infestation on *Zea mays* stimulates root exudation of a benzoxazinoid breakdown compound 6-methoxy-benzoxazolin-2-one (MBOA). This metabolite enhances microorganisms in the rhizosphere that increase plant resistance against these caterpillars (Hu et al. 2018). Beneficial microorganisms can induce or prime plant defence in systemic tissues via various pathways in an SA- or JA/ethylene-dependent or independent manner (Niu et al. 2011; Pieterse et al. 2014; van de Mortel et al. 2012). Microbe-induced systemic resistance (ISR) is a mechanism where rhizosphere microorganisms increase resistance to insects via for instance the JA and ethylene pathways. These pathways can increase production of (toxic) secondary metabolites that may reduce the performance of caterpillars (Pangesti et al. 2016; Yang et al. 2025).

In this study, we investigated herbivory-induced PSF in cabbage, *Brassica oleracea*, and the factors governing PSF outcomes. We investigated whether caterpillar-induced changes in the rhizosphere of two accessions, a wild and a cultivated cabbage accession, enhanced plant defence against *M. brassicae* caterpillars. This was tested at three different caterpillar densities, different feeding times and in three different soil types to reveal the key factors that influence PSF. We studied a wild and a cultivated *B. oleracea* accession that were expected to have a different level of resistance to chewing herbivores as shown for similar wild and cultivated *B. oleracea* accessions (Gols et al. 2008a, b). It was hypothesised that *M. brassicae* on the wild cabbage accession induces a stronger PSF-mediated plant defence against *M. brassicae* compared to the cultivated cabbage accession. We refer to increased plant resistance as a positive PSF response. Furthermore, we expected that the strength of PSF increases with intensity of herbivory in receiver plants, as more feeding damage may stimulate plants to invest extra resources in the interactions with a beneficial soil microbiome (Schultz et al. 2013; Xing et al. 2024). Furthermore, we expect that PSF varies between soil types because different soils have distinct chemical and microbial compositions (Bai et al. 2022; Gfeller et al. 2023; Wagner and Mitchell-Olds 2023).

## Materials and methods

### Plants

In our study, we used uncoated seeds of cultivated white cabbage plants (*Brassica oleracea* L. var. *capitata* cv Rivera, hereafter called Rivera) provided by Bejo Seeds (Warmerhuizen, the Netherlands), and a wild cabbage (*B. oleracea*) accession (hereafter called Durdle Door) originally collected as seeds in Durdle Door (Dorset, UK) (Gols et al. 2008b). Seeds were surface sterilised by washing them for one minute in 80% ethanol followed by one to three minutes in 1% sodium hypochlorite with 0.05% Tween 20 to lower surface tension. Between and after these steps, seeds were washed with autoclaved tap water. Next, they were stratified in the dark at 4 °C for three days and then left on humid filter paper in a Petri dish to acclimate at room temperature for one day before sowing. Plants were grown in a greenhouse at 23 ± 2 °C, 50–60% relative humidity and a 16:8 light:dark regime. Seeds were sown in seedling trays with soil, except for the feedback phase of experiments 2 and 3 where seeds were directly sown in 1-L pots (as described below). Afterwards, seedling trays and pots were covered with punctured, transparent plastic foil to increase humidity. Foil was removed when cotyledons unfolded. Seedlings were transferred from trays to 1-L pots after six days. They were grown in a complete randomised block design with one plant per treatment in each block, except for experiment 4, which had multiple plants per block. Plants were watered from below via dishes underneath each pot once or twice a week with 50 mL tap water depending on plant needs. In addition to watering, we fertilised with 50 mL ½-strength Hoagland once a week when plants were younger that two months and twice a week when plants were older than two months.

### Soil

“MiCRop” soil was used for all experiments. This is a loamy sandy soil (pH: 5.2; organic carbon: 1.6%; Table S1, S2; Fig. S1) collected from an organically managed agricultural field at Nergena, Bennekom, the Netherlands (coordinates: 51.996250° N, 5.659375° E). It was collected in 2014 by excavating the 80-cm top layer of the field, and was subsequently stored outdoors and left unmanaged, thereby ensuring that a natural soil community was sustained through growth of wild plants. Before the start of experiments, batches of soil were air-dried and sieved through a 5-mm mesh to remove bigger particles and roots and stored in the dark until use in the experiments. At the start of the experiment, water content of the soil was gradually increased by mixing soil with 100 mL of tap water per kg of soil. In addition, seedling trays were placed in trays with water until the soil surface was moistened. Pots received 100 to 150 mL of tap water to each pot just after sowing and transplantation.

As a second soil, “Reijerscamp” soil was used in experiment 3 (see below) because it has been shown to be a disease-suppressive soil (Berendsen et al. 2018). This sandy loam soil (pH: 4.9; organic carbon: 3.9%; Table S1, S2; Fig. S1) was collected from the nature reserve Reijerscamp, Wolfheze, the Netherlands (coordinates: 52.010255° N, 5.779983° E). This nature reserve was created from a conventional agricultural field in 2000 (Berendsen et al. 2018) by removing 80 to 100 cm of top soil to stimulate nutrient-poor vegetation development (Bosscher 2024). In September 2023, soil for experiment 3 was collected by taking the top 20 cm after removing the vegetation. The soil was air dried and sieved through a 6 mm-mesh to remove big particles and plant materials and was stored in the dark until further use and processed in the same way as described for “MiCRop” soil.

### Insects

In all experiments, we used first instar larvae (L1) of *Mamestra brassicae* L. (Lepidoptera: Noctuidae), which were reared at 23 ± 2 °C, 50–60% relative humidity and a 16:8 light:dark regime on *B. oleracea* L. var. *gemmifera* (cv Cyrus, Hey Melis or Cobelius). Synchronised eggs of *M. brassicae* were collected and incubated at room temperature, and newly hatched larvae were transferred to the youngest fully expanded leaf using a fine brush. Each plant was contained in a mesh bag, and caterpillars could move freely within this bag. Control plants that were left non-infested were contained in mesh bags in the same way. After caterpillar infestation, *M. brassicae* performance was assessed by caterpillar survival, caterpillar weight and leaf damage. To determine caterpillar weight, we placed caterpillars in Petri dishes on ice before weighing them individually on an analytical balance (d = 0.001 mg). Leaf damage was assessed in experiments 1 and 2 by image analysis. First, leaves were detached from the plant and placed on a Nanlite Compact 24 LED photo light (NanGuang, Shantou, China) to create high contrast. Photos were then taken with a smart phone and analysed with LeafByte 1.3.0 providing total leaf area and leaf area eaten (Getman‐Pickering et al. 2020).

### Host-plant resistance

Before performing PSF experiments, the resistance of Durdle Door and Rivera plants to *M. brassicae* caterpillars was compared by assessing caterpillar survival, individual caterpillar weight, leaf damage, total leaf area and root dry weight (Table S3). Root dry weight was determined by washing the soil from the roots, oven drying the roots for 2 days at 105 °C, and weighing dry material on a weighing scale (d = 0.1 mg).

### General procedures plant-soil feedback experiments

#### Conditioning phase

**Fig. 1:**
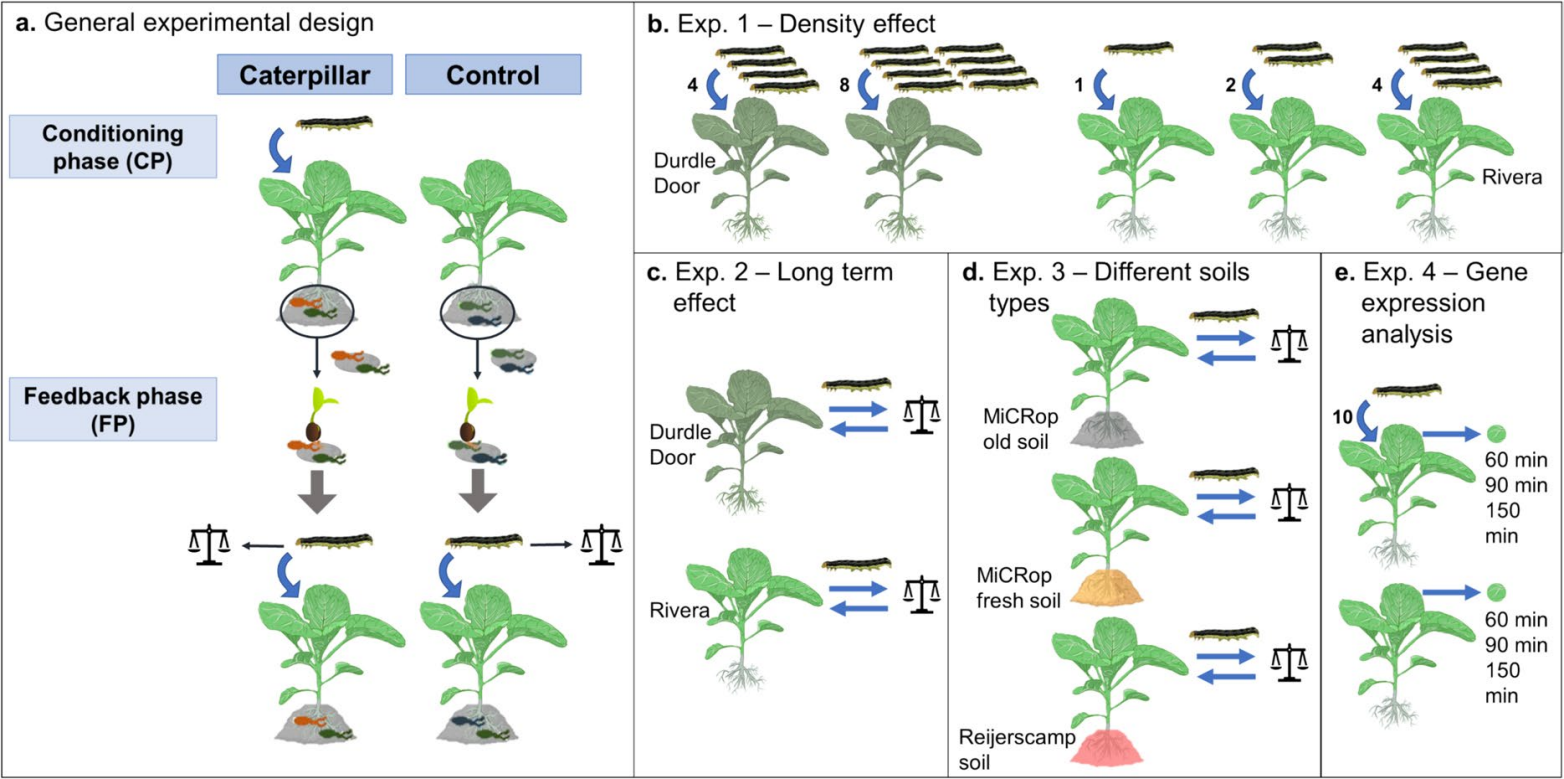
(**a**) General experimental design of PSF experiments using a cabbage cultivar (Rivera) and a wild *Brassica oleracea* accession (Durdle Door). In the conditioning phase (CP), MiCRop soil was conditioned by plants with *M. brassicae* caterpillars (caterpillar-conditioned soil) or by plants without herbivore infestation (control-conditioned soil). Conditioned rhizosphere and bulk soil of both treatments were collected, kept separate and used for a new generation of plants in the feedback phase (FP). Plants of both treatments were infested with *M. brassicae* caterpillars in the FP to assess the effect of soil conditioning on plant resistance. (**b-e**) Specific modifications per experiment (Exp.). (**b**) Exp. 1: To test how the intensity of herbivory affects PSF, different insect densities were applied in the FP, where Durdle Door received 4 or 8 caterpillars and Rivera 1, 2 or 4 caterpillars per plant. Caterpillar densities were based on the relative resistance of each accession. (**c**) Exp. 2: To study temporal PSF dynamics, caterpillars were weighed and transferred back to their host plant several times in the FP. (**d**) Exp. 3: To investigate the role of soil type, Rivera plants were grown on three different soil types, and caterpillars were weighed and transferred back on the plant several times in FP. (**e**) Exp. 4: To assess whether PSF affected induced plant defences, leaf discs of Rivera plants were sampled 60, 90 and 150 min after the start of caterpillar infestation in the FP for gene expression analysis. Non-infested plants were used to measure constitutive expression. Unique plants were sampled for each time point

To create a soil-borne legacy, we allowed L1 caterpillars to feed on half of the plants for 10 to 14 days depending on the experiment, and left half of the plants non-infested as a control (Fig. 1a; Table S3). The relatively resistant Durdle Door plants received more caterpillars than Rivera plants to obtain comparable damage levels on both accessions. This results in similar levels of stress making caterpillar-induced PSF comparable between different accessions. After 10 to 14 days, caterpillars were removed, and bulk and rhizosphere soil were collected and kept separate per soil-conditioning treatment to be used as inoculum in the feedback phase. Bulk soil was defined as the soil that falls off the roots after removing the pot and gently shaking the roots. Rhizosphere soil was collected subsequently by roughly shaking and rubbing the roots. The collected soil was air-dried if needed and sieved through a 0.5 or 1-cm mesh. A sieve with a mesh size of 1.6 cm was used for air-dried bulk soil. Both soils were stored at 4 °C in the dark until further use. In addition to these soils, rhizoplane soil was obtained by washing roots in autoclaved tap water and collecting the soil residue that was airdried in a fume hood overnight.

#### Feedback phase

We assessed the PSF of soil from the conditioning phase on a new set of plants and caterpillars (Fig. 1a). The conditioned soils from different treatments were kept separate and always matched the treatment in the feedback phase. No cross inoculation occurred between different accessions or soil types. Depending on the experiment, we used one of the following methods for soil inoculation: soil slurry transfer, dry rhizosphere transfer, or bulk soil transfer (Table S3). Three different methods were used in this study as the soil inoculation procedures were optimised over time to maximise the PSF effect of the rhizosphere. Soil-slurry was made the day after rhizosphere collection by mixing the rhizosphere and rhizoplane soil in a 10 mM KH_2_PO_4_ buffer in a ratio of 1:9 v/v. Seeds were dipped in the soil slurry for 1 min and subsequently sown in 1-L pots filled with a soil mixture of soil slurry and γ-irradiated soil in a ratio of 1:9 m/m (γ-irradiation dose was > 8.3 kGy). Water content of this soil mixture was increased by adding tap water (1:10 tap water:soil m/m). For the dry rhizosphere transfer method, γ-irradiated soil was pre-wetted with tap water (10:1 m/m), before it was extensively mixed with dry rhizosphere soil (9:1 m/m). The rhizosphere soil mixture was added to seedling trays, and seeds were sown. Seedlings were transferred after nine days to 1-L pots with a 1:1 m/m mix of dry bulk soil from the conditioning phase and pre-wetted γ-irradiated soil (10:1 soil:tap water m/m). For bulk soil transfer, γ-irradiated soil was mixed 1:1 m/m with bulk soil from Durdle Door plants after the conditioning phase of experiment 1. Water content of this soil mixture was increased by adding tap water (1:10 tap water:soil m/m). For all soil inoculation methods, caterpillars were introduced on the plants after 22 to 73 days of plant growth (Table S3) to assess the soil-conditioning effect on caterpillar performance as described above.

### Experiment 1: Density effect

In this first PSF experiment, different densities of caterpillars were placed on the plant in the feedback phase to investigate the effect of caterpillar-induced PSF on different densities of *M. brassicae* (Fig. 1b, Table S3). We assessed *Mamestra brassicae* survival, individual caterpillar weight, leaf damage, total leaf area and root dry weight after 10 days of caterpillar infestation. Root dry weight was measured at a scale (d = 0.1 mg) after washing roots from the soil and oven drying for 3 days at 70 °C.

### Experiment 2: Effect of infestation time

To assess temporal dynamics of PSF over a longer infestation period, we first performed a detailed time trend analyses on Durdle Door (Fig. 1c; Table S3). On each plant, two L1 caterpillars were introduced, and seven days later, both caterpillars were weighed individually. One of the two caterpillars was placed back on the same plant when both caterpillars survived. Then, individual caterpillar weight was assessed after 14, 21 and 24 days. After weighing, caterpillars were returned to the same plant. To observe whether the weekly transfers affected caterpillar performance, a batch of caterpillars grown on plants in control-conditioned soil were left untouched and were weighed only once after 21 days. We did not find an effect of handling caterpillars on their weight (Mann–Whitney U Test: W = 82, p = 0.598).

To validate temporal PSF dynamics, *M. brassicae* performance was subsequently studied on both Durdle Door and Rivera plants during a long-term infestation (Fig. 1c; Table S3). In the feedback phase, caterpillars on Rivera plants were weighed after 17 days and caterpillars on Durdle Door plants after 18 and 25 days. Caterpillars were returned to the same plant after weighing. No caterpillar weight measurements were taken at a second time point for Rivera plants as caterpillars were already pupating in the week after the first measurements. Caterpillars were allowed to pupate in the soil of their respective pots. Emerging moths were collected to assess survival.

### Experiment 3: Different soil types

To test the uniformity of PSF across soils of different compositions and storage treatments, we tested caterpillar-induced PSF in three soil types by growing Rivera plants in the conditioning phase on: (1) an old batch of “MiCRop” soil (stored for six months), (2) a new batch of “MiCRop” soil (freshly collected just before the experiment) and (3) “Reijerscamp” soil (Fig. 1d; Table S3). In the feedback phase, neonate *M. brassicae* larvae were feeding on *B. oleracea* L. var. *gemmifera* cv Cobelius for two days before caterpillars were introduced on 73-day-old experimental Rivera plants. Two-day-old larvae could penetrate the wax layer of 73-day-old plants while neonates could not. Each experimental plant was exposed to two caterpillars. Two days later, one of the two caterpillars was removed when both survived. Individual caterpillar weight was measured 7, 13, 17 and 19 days after hatching of the eggs. Caterpillars were returned to the same plant after weighing. Fourteen days after the start of infestation, a fire outbreak elsewhere resulted in smoke entering the greenhouse compartment on that day but no abnormalities were observed in the experiment.

### Experiment 4: Gene expression analysis

#### Plant growth and leaf sampling

Finally, we investigated the effect of PSF on defence gene expression in the feedback phase by infesting 30 plants per soil treatment with 10 neonate caterpillars which were starved upon emergence from eggs for half a day (Fig. 1e; Table S3). Their movement was restricted to the youngest fully expanded leaf by wrapping cotton wool around the petiole. The other 30 plants per soil treatment were treated the same way but were not infested with caterpillars. Plants were evenly and randomly divided over three sampling time points (60, 90 and, 150 min post infestation). At 60 min, we observed the first leaf damage caused by the caterpillars on each plant. At the three time points, leaf samples were taken by removing the leaf from the plant, removing the caterpillars if present, and taking twelve leaf discs with a diameter of 0.5 or 0.6 cm close to the feeding sites using a cork borer. Non-infested plants were sampled at the same time point. Each biological replicate consisted of leaf discs from one plant. Samples were directly flash frozen in liquid nitrogen and used later for RNA extraction and real-time quantitative PCR (RT-qPCR) analysis.

#### Gene expression analysis

For RT-qPCR, we homogenized the leaf samples in cold blocks in a TissueLyser II (Qiagen, Hilden, Germany) with three glass beads (3 mm) per tube for 1:30 min. Afterwards, RNA was extracted with the Isolate II Plant RNA kit (Bioline, London, UK) according to the protocol of the manufacturer. Centrifuge steps were doubled compared to the protocol. Then, cDNA was synthesized from RNA with SensiFAST cDNA synthesis kit (Bioline, London, UK) according to the protocol of the manufacturer and diluted five times. Next, gene expression was measured on JA-biosynthesis gene *AOS*, JA-response genes *MYC2* and *PDF1.2*, indole-glucosinolate-biosynthesis gene *CYP81F4*, aliphatic-glucosinolate-biosynthesis gene *MYB28*, SA-biosynthesis gene *ICS1*, and SA-response gene *BGL2* (Table S4) by RT-qPCR with the qPCR SensiFast SYBR Green kit (Bioline, London, UK) on a Bio-Rad CFX96 Real-Time PCR Detection System machine (Bio-Rad, California, USA). Three interrun calibrators, each consisting of a mix of 40 different samples, were used to correct for interrun variation. *PER4* and *SAR1a* (Table S4) were the most stably expressed reference genes after a selection by RT-qPCR and GeNorm analysis in qbase + 3.4 (Biogazelle, Gent, Belgium) out of *ACT2*, *BTUB*, *GAPDH*, *EF1a*, *PER4* and *SAR1a*-reference genes (Vandesompele et al. 2002). Raw Cq values from the genes of interest were processed in qbase + 3.4 (Biogazelle, Gent, Belgium) using the 2^−ΔΔct^ (Livak and Schmittgen 2001) and Pfaffl methods (Pfaffl 2001) to calculate relative gene expression and correct for primer efficiency (Table S4). *MYB28 and PDF1.2* had high primer efficiency and should be interpreted with caution (Table S4). Differences between runs were corrected for with interrun calibrators. During subsequent statistical analysis, two samples for *AOS* were left out as they had unrealistically high relative gene expression (RGE) (CTRL soil – non-infested – 150 min: RGE = 21.84; CTRL soil – caterpillar infestation – 150 min: RGE = 9.37), but this did not affect the conclusion.

#### Statistics

Statistical analyses were performed in R 4.4.1 (R-Core-Team 2024). Data were explored using plyr (v1.8.9; Wickham 2011), and analysed with two-sample t-test, Mann Whitney-U test or (generalised) linear (mixed) models ((G)L(M)Ms) using glmmTMB (v1.1.9; Brooks et al. 2017) (Table S5). The best model was chosen based on QQ-plots, Levene tests, residual plots and model assumptions with DHARMa (v0.4.6; Hartig 2022) with analysis conducted on transformed data and on model scale where applicable. For (G)L(M)Ms, statistical information of the main effects was obtained with the Anova function of the Car package (v3.1.2; Fox and Weisberg 2019) and Tukey post-hoc tests were performed with emmeans (v1.10.4; Lenth 2024), multcomp (v1.4.26; Hothorn et al. 2008) and multcompView (v0.1.10; Graves et al. 2024). For mixed models, block (location in the greenhouse compartment) was included as a random factor. In addition, the random factor Plant ID was included in caterpillar weight analysis when multiple caterpillars were feeding from the same plant. Random factors were left out if they seriously reduced the model fit. In only one case (caterpillar weight on Rivera after 17 days, Fig. 4c), we could not avoid heteroscedasticity, but validated the significant outcome of the best-fitting GLMM with a Mann Whitney-U test. Caterpillar growth rate was calculated from caterpillar weight data with the slope function in Microsoft Excel (Microsoft 365 MSO Version 2402). Only caterpillars with data on each time point were included. For experiment 2, we took the average weight of the caterpillars per plant at 7 dpi, and the exponential growth rate of the first three time points (exponential phase) was calculated on ln-transformed caterpillar weight data. Linear caterpillar growth rate was calculated on the first three time points (linear phase) of caterpillar weight data of experiment 3. We used ggplot2 (v3.5.1; Wickham 2016), ggpattern (v1.1.1; FC et al. 2024) and ggpubr (v0.6.0; Kassambara 2023) for graphical presentation of the data. Data were always presented in its raw form. In line plots, we used geom_smooth with default settings “method = 'loess'” and “formula = 'y ~ x'” with 95% confidence interval. In all experiments, PSF effects were interpreted in terms of plant resistance against caterpillar feeding. These effects were considered positive when caterpillar performance was higher on control- than on caterpillar-conditioned soil and negative when caterpillar performance was lower on control than on caterpillar-conditioned soil. No effects between soil-conditioning treatments were interpreted as neutral PSF.

## Results

### Host-plant resistance is higher in wild accession than in cultivated relative

**Fig. 2:**
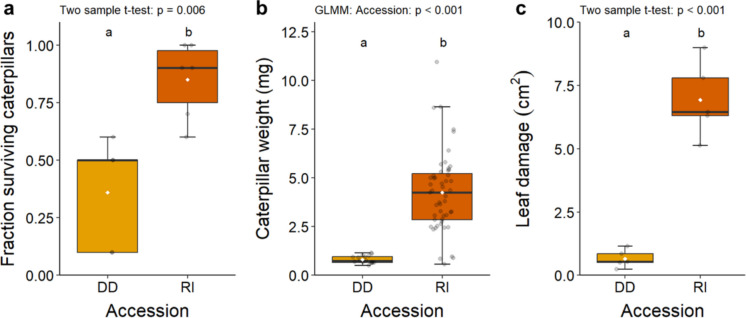
A comparison of (**a**) fraction surviving *M. brassicae* caterpillars (DD: n = 5; RI: n = 6), (**b**) individual caterpillar weight (DD: n = 17; RI: n = 57) and (**c**) leaf damage (DD and RI: n = 5) on Durdle Door (DD) and Rivera (RI) plants. Boxes in the boxplots represent the second and third quartile of the data, whiskers of the boxplots represent at maximum 1.5 times the distance between the first and third quartiles, grey dots display single data points, and white diamonds indicate mean per treatment. Letters above boxplots present statistical differences between treatments based on a Tukey post-hoc test (α = 0.05)

Before assessing PSF, we compared host-plant resistance to *M. brassicae* in the wild accession Durdle Door and the cultivated accession Rivera. We measured caterpillar survival, caterpillar weight and leaf damage after five days of *M. brassicae* caterpillar infestation. Caterpillar survival was more than two times higher on Rivera plants than on Durdle Door plants (two-sample t-test: t = 3.86, df = 6.88, p = 0.006; Fig. 2a). This low survival on Durdle Door was likely caused by cannibalism (Richter 1990) as no traces of missing caterpillars were left on the plant or in the soap water around the pots. In addition, caterpillars on Rivera plants were five times heavier than those on Durdle Door plants (GLMM: χ^2^ = 119.7, df = 1, p < 0.001; Fig. 2b). Leaf damage was more than 10 times higher on Rivera plants (two-sample t-test: t = 9.17, df = 4.45, p < 0.001; Fig. 2c). More specifically, *M. brassicae* consumed, on average, 5.9% of available leaf material on Rivera and 0.7% on Durdle Door plants. These larvae clearly did not accept Durdle Door as a host as they created many small feeding sites on Durdle Door and were observed to climb into the net around the plant. In contrast, the herbivores on Rivera plants stayed on their host plant and created only few feeding sites with large areas of removed foliage. In addition to caterpillar performance, plant traits were measured as well. Total leaf surface area of uninfested plants was comparable between accessions (two-sample t-test: t = 1.65, df = 6.41, p = 0.147; Fig. S2a). In contrast, root biomass of Rivera plants was 67% higher than of Durdle Door plants (two-sample t-test: t = 4.46, df = 6.35, p = 0.004; Fig. S2b).

### Density-dependent PSF differs between wild and cultivated cabbage

A PSF experiment was performed to study if soil-mediated effects on insect and plant performance differed between the wild cabbage Durdle Door and the cultivar Rivera. These soil-borne effects were tested by growing plants on soil that was conditioned by plants exposed to *M. brassicae* caterpillars or control conditions. We introduced different numbers of *M. brassicae* to assess whether a soil-borne legacy can result in different outcomes when the next generation of plants experiences different intensities of herbivory. The soil-conditioning treatment did not affect total leaf area or root biomass for both Rivera and Durdle Door (Fig. S3). For Rivera, higher caterpillar density and thus more feeding resulted in a 8% decrease in total leaf area (LM: χ^2^ = 7.73, df = 1, p = 0.021; Fig. S3b), while for Durdle Door only root biomass declined (28%) with increasing caterpillar density (χ^2^ = 4.53, df = 1, p = 0.033; Fig. S3c).

**Fig. 3:**
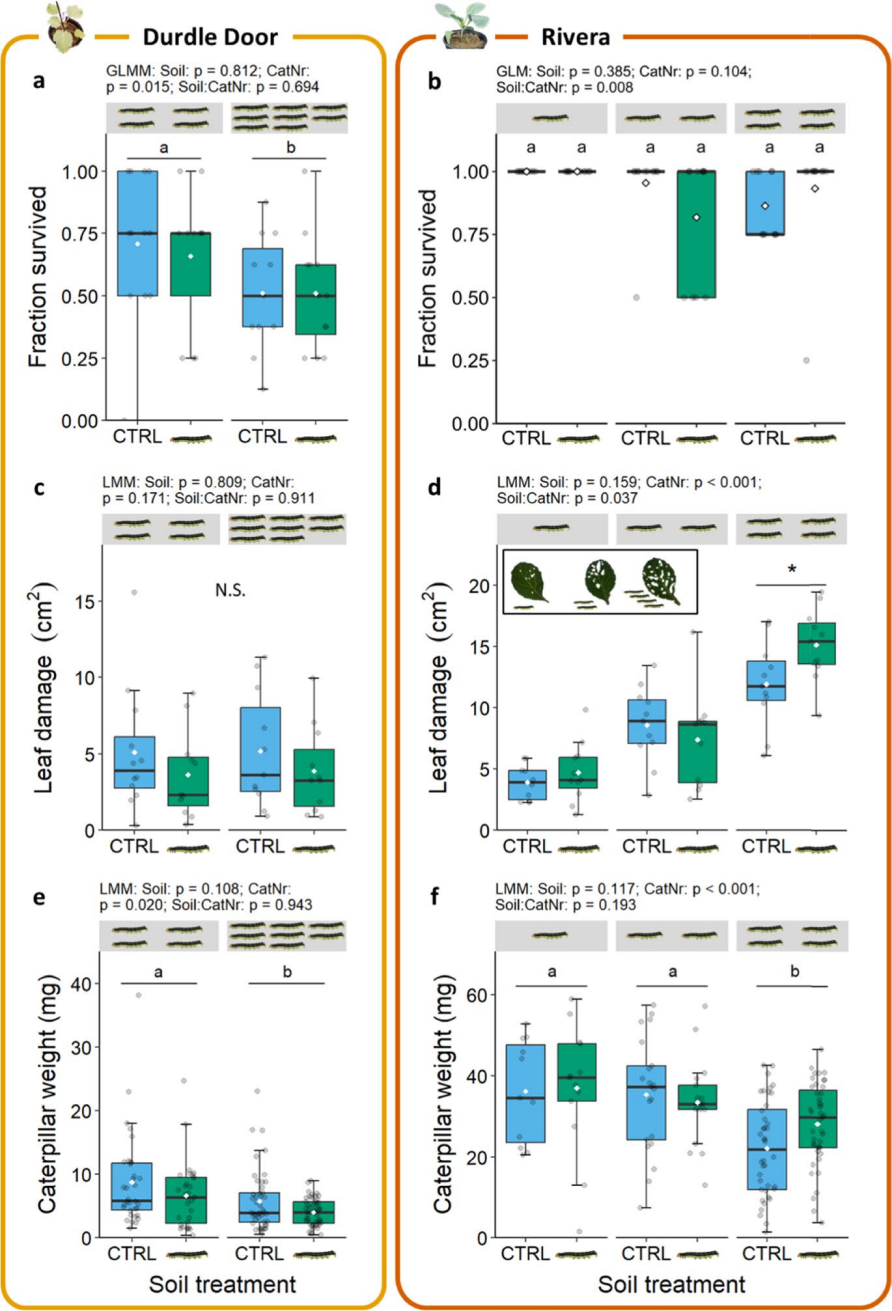
Effects of soil legacy in control-conditioned (CTRL) or caterpillar-conditioned (  ) soil on (**ab**) fraction surviving *M. brassicae* caterpillars, (**cd**) individual caterpillar weight and (**ef**) total leaf damage on Durdle Door (left panels) and Rivera (right panels) plants at different *M. brassicae* densities (CatNr) in the feedback phase (Experiment 1). Caterpillars were allowed to feed for 10 days. Densities are indicated by the number of caterpillars (  ) above the boxplots (Durdle Door: 4 and 8 caterpillars; Rivera: 1, 2 and 4 caterpillars). Fraction survived: n = 11–12; caterpillar weight: n = 11–49; consumed leaf area: n = 10–12. The panel in (**d**) shows leaves with representative damage per caterpillar density. Boxes in the boxplots represent the second and third quartile of the data, whiskers represent at maximum 1.5 times the distance between the first and third quartiles, grey dots display single data points, and white diamonds indicate mean per treatment. Letters above boxplots present statistical differences between treatments based on a Tukey post-hoc test (α = 0.05). An asterisk indicates a significant difference between two specific treatments using the same test. N.S. means no statistical differences

With respect to caterpillar performance, caterpillar survival on Durdle Door plants differed between caterpillar densities (GLMM: χ^2^ = 5.87, df = 1, p = 0.015; Fig. 3a) with 69% survival on plants with 4 caterpillars and 51% on plants with 8 caterpillars. No effect was found of soil conditioning on caterpillar survival (GLMM: χ^2^ = 0.057, df = 1, p = 0.812), the amount of leaf damage (LMM: χ^2^ = 1.87, df = 1, p = 0.171; Fig. 3c), or *M. brassicae* weight (LMM: χ^2^ = 2.58, df = 1, p = 0.108; Fig. 3e). Furthermore, caterpillar density did not influence leaf damage (LMM: χ^2^ = 0.059, df = 1, p = 0.809; Fig. 3c), but caterpillar weight decreased by 37% when caterpillar density increased (LMM: χ^2^ = 5.43, df = 1, p = 0.020; Fig. 3e).

Caterpillar survival on Rivera plants (93%) was higher than on Durdle Door plants (60%), and no differences between caterpillar densities or soil conditioning treatments were observed (GLMM density: χ^2^ = 4.53, df = 2, p = 0.104; GLMM soil: χ^2^ = 0.753, df = 1, p = 0.385; Fig. 3b). Total leaf damage in Rivera was affected primarily by actual caterpillar density (LMM: χ^2^ = 116.4, df = 2, p < 0.001; Fig. 3d), but also by soil conditioning when plants were exposed to the highest caterpillar density (LMM: χ^2^ = 6.58, df = 2, p = 0.037; Fig. 3d). At this density, leaf damage was 27% higher on caterpillar-conditioned soil compared to control-conditioned soil (p = 0.009). *Mamestra brassicae* weight decreased when more caterpillars were feeding on a plant (LMM: χ^2^ = 26.9, df = 2, p < 0.001; Fig. 3f), and showed a non-significant 27% increase when their host was grown on caterpillar-conditioned soil (LMM: χ^2^ = 3.30, df = 2, p = 0.193; Fig. 3f). Overall, our findings show that PSF differs between cabbage accessions and depends on herbivore intensity. We found no PSF effect on Durdle Door and increased feeding damage on the heavily-infested Rivera cultivar when soils were conditioned by previous herbivory.

### PSF effects increase with longer infestation time

**Fig. 4:**
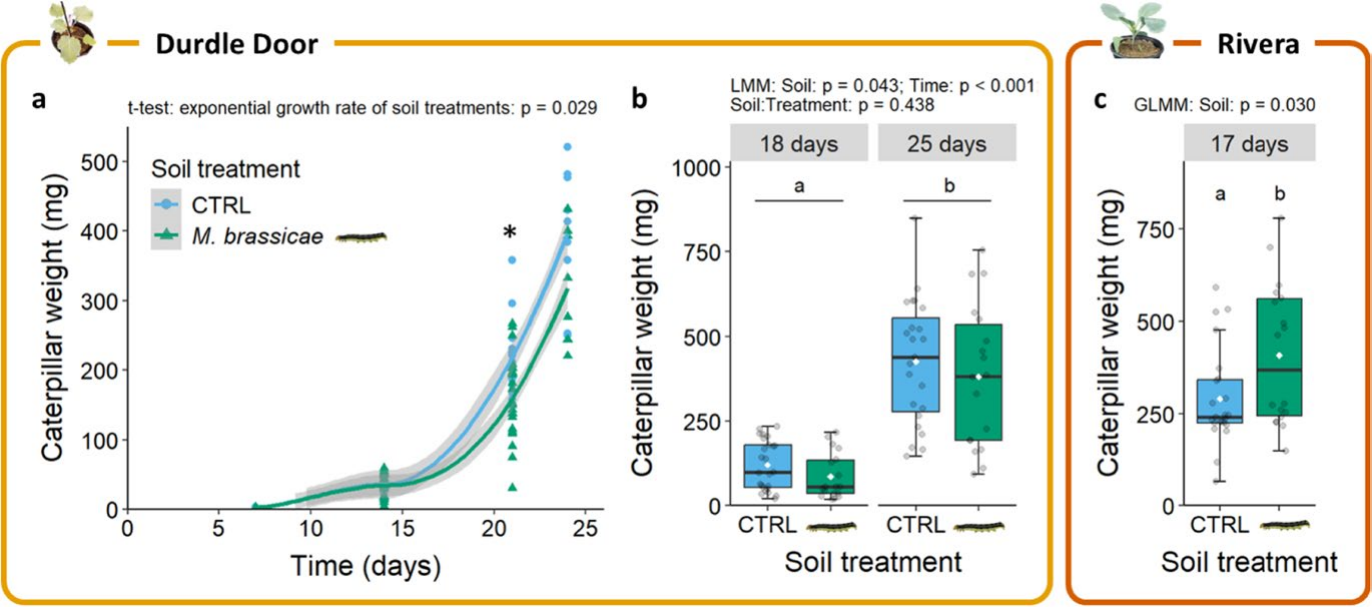
Weight of individual *M. brassicae* caterpillar over time in the feedback phase of two different PSF experiments where plants were growing on control- (CTRL) or caterpillar-conditioned (  ) soil (Experiment 2). (**a**) *Mamestra brassicae* caterpillar weight on Durdle Door plants growing in conditioned soils (CTRL: n = 17; *M. brassicae*: n = 24). (**b** and **c**) *Mamestra brassicae* weight on different time points after infestation in the feedback phase of a second PSF experiment with (**b**) Durdle Door and (**c**) Rivera (CTRL: n = 23–25; caterpillar-conditioned soil: n = 18). Boxes in the boxplots represent the second and third quartile of the data, whiskers represent at maximum 1.5 times the distance between the first and third quartiles, dots in line- and boxplots display single data points, and white diamonds indicate mean per treatment. The star above data points indicates a statistical difference between treatments based on a t-test (α = 0.05), and letters above boxplots present statistical differences between treatments based on a Tukey post-hoc test (α = 0.05)

Since we observed a small effect of caterpillar-induced PSF on plant resistance in Rivera but not in Durdle Door plants after 10 days of caterpillar infestation, we hypothesised that a longer infestation time may also reveal a PSF effect in Durdle Door. When examining PSF over a longer time frame, caterpillar survival was relatively high on Durdle Door compared to the previous experiments (> 80%) and was similar for both soil-conditioning treatments. However, the growth rate of *M. brassicae* was 10% lower on caterpillar*-*conditioned than on control-conditioned soil (two-sample t-test: t = 2.27, df = 39.0, p = 0.029; Fig. 4a). This difference was observed only after 21 days of feeding with a 20% reduction in caterpillar weight at that day (Mann Whitney-U test on caterpillar weight at 21 days: W = 315, p = 0.002).

In a separate experiment, this decrease in caterpillar weight was confirmed on Durdle Door plants grown in caterpillar-conditioned soil after 18 and 25 dpi (28% and 11% reduction, respectively; LMM: χ^2^ = 4.09, df = 1, p = 0.043; Fig. 4b). In contrast, caterpillars on Rivera plants developed much faster and were 40% heavier on plants grown in caterpillar-conditioned soil (GLMM: χ^2^ = 4.68, df = 1, p = 0.030; Fig. 4c), confirming our previous results of PSF-induced susceptibility in Rivera. Overall, 93% of the caterpillars developed into moths after feeding on Rivera and 27% after feeding on Durdle Door, irrespective of soil treatment. The remaining caterpillars did not start pupation and died.

### Consistent PSF across different soil types

**Fig. 5:**
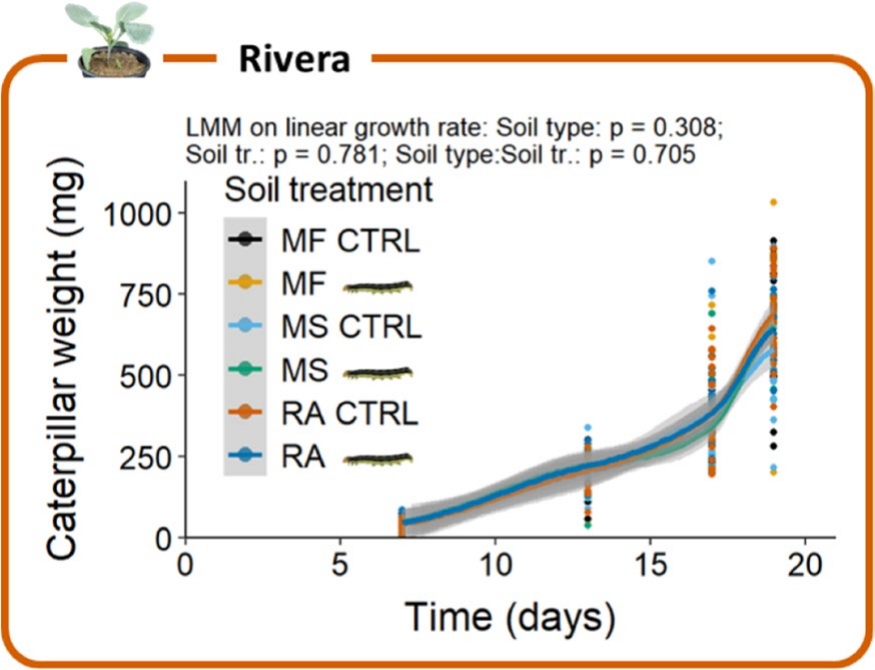
*Mamestra brassicae* caterpillar weight at different time points in the feedback phase of a PSF experiment on Rivera with different soil types: freshly collected “MiCRop” soil (MF), “MiCRop” soil that was stored for half a year (MS) and “Reijerscamp” soil (RA). Each plant grew on control- (CTRL) or caterpillar-conditioned (  ) soil treatment (soil tr.) (Experiment 3). Caterpillars were weight individually (n = 19 or 20). Dots display single data points

As soil type and longer-term soil legacy are important factors in PSF (Hannula et al. 2021; Kardol et al. 2013; van der Putten et al. 2013), we tested Rivera for caterpillar-induced PSF in soils from different origins and different storage regimes. In addition to the “MiCRop” soil (both freshly collected and stored for half a year), we selected the disease-suppressive “Reijerscamp” soil (Berendsen et al. 2018). In the feedback phase, we did not observe any differences in caterpillar growth rate between the three soil types (LMM: χ^2^ = 2.36, df = 2, p = 0.308; Fig. 5), and caterpillar weight was not affected by conditioning of the soil with or without *M. brassicae* (soil-conditioning treatment) either (LMM: χ^2^ = 0.077, df = 1, p = 0.781; Fig. 5).

### PSF downregulates the JA pathway

**Fig. 6:**
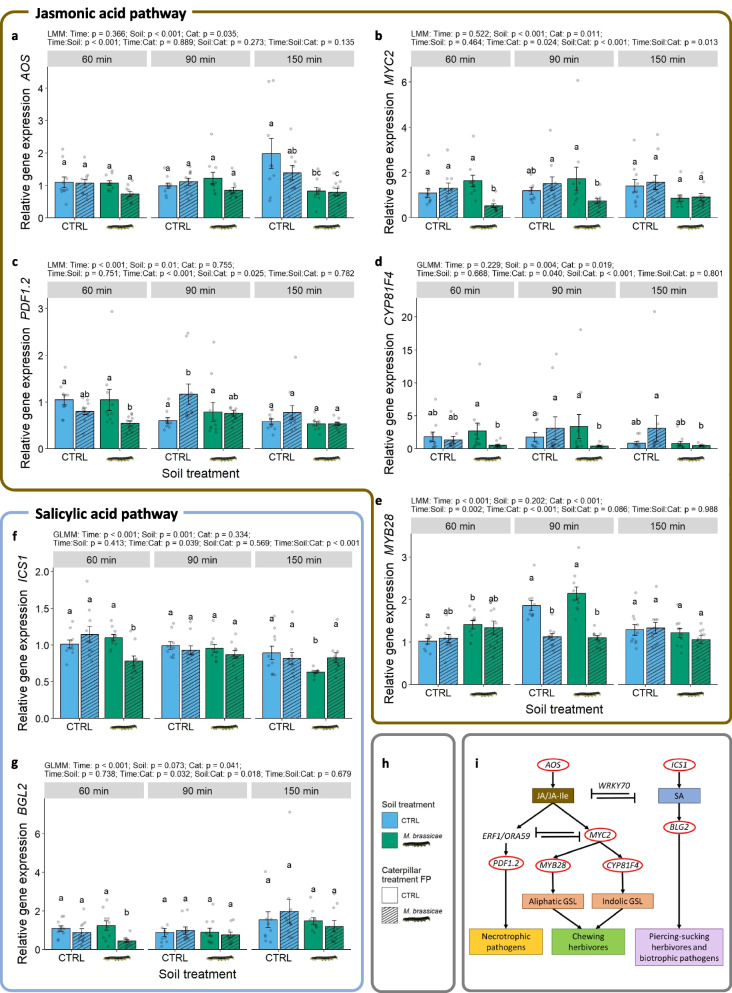
Effect of soil-conditioning treatment and *M. brassicae* herbivory on relative expression of genes involved in the JA (brown panel) and SA pathways (blue panel) (Experiment 4). Relative gene expression of (**a**) *AOS*, (**b**) *MYC2*, (**c**) *PDF1.2*, (**d**) *CYP81F4*, (**e**) *MYB28*, (**f**) *ICS1* and (**g**) *BGL2* was measured at 60, 90 and 150 min after *M. brassicae* introduction (Time) in the feedback phase of a PSF experiment where plants were growing on caterpillar-conditioned (  ) or control-conditioned (CTRL) soil (Soil) and with (*M. brassicae*) or without (CTRL) caterpillar infestation (Cat; **h**) (n = 10). The tested genes play a role in the JA and SA biosynthesis and downstream signalling pathways shaping the defence against pathogens and insects. (**i**) Display of a simplified gene network in which the tested genes are highlighted by red circles (Ali et al. 2024; Karssemeijer et al. 2022a; Pangesti et al. 2016; Verma et al. 2025; Wasternack and Hause 2013; Wasternack and Strnad 2019). Error bars represent the standard error, and grey dots display single data points. Letters above bar plots represent statistical differences between treatments based on a Tukey post-hoc test (α = 0.05)

In the first experiments, we observed increased caterpillar performance and feeding damage in Rivera plants due to caterpillar-induced PSF. To investigate whether this was caused by increased susceptibility and to understand the underlying PSF mechanisms, we analysed defence gene expression in the JA and SA biosynthesis and signalling pathways (Fig. 6i) in the feedback phase of a PSF experiment. We succeeded in developing primers with fair efficiencies, apart from *MYB28 and PDF1.2* (130–140%, Table S4), but included all primer pairs for a broad view on responses in the JA signalling pathway. The first leaf samples were collected 60 min after *M. brassicae* introduction when the first feeding damage was observed on all plants. Contradictory to the general concept that chewing-herbivore feeding activates JA signalling (Erb and Reymond 2019; Howe and Jander 2008), we did not observe herbivory-induced upregulation of JA biosynthesis (*AOS*; Fig. 6a) or downstream JA-signalling genes (*MYC2*, *PDF1.2* and *CYP81F4*; Fig. 6b, c, d; Table S6). Furthermore, SA biosynthesis (*ICS1*) and downstream signalling of this hormone (*BGL2*) were not strongly affected (Table S6). However, caterpillar-conditioned soil reduced *AOS* expression after 150 min compared to control-conditioned soil with (79% lower; p = 0.002) and without caterpillar feeding (61% lower; p = 0.021) (Fig. 6a). *MYC2*, *PDF1.2*, *CYP81F4*, *ICS1* and *BGL2* were downregulated as well on caterpillar-conditioned soil but only when caterpillars were feeding in the feedback phase (29–87% reduction). This occurred after 60 min for *PDF1.2*, *ICS1* and *BGL2* (Fig. 6c, f, g) and after 60 and 90 min for *MYC2* and *CYP81F4* (Fig. 6b, d), and these effects disappeared at later time points. Only *ICS1* remained downregulated at 150 min for caterpillar-conditioned soil treatment without caterpillars in the feedback phase (p = 0.001) compared to control-conditioned soil, but this effect was counteracted by caterpillar feeding (p = 0.013; Fig. 6f).

We observed a higher constitutive expression of the aliphatic biosynthesis transcription factor gene *MYB28* on caterpillar-conditioned soil compared to control-conditioned soil after 60 min (no herbivory: 49% increase, p = 0.025; Fig. 6e). In contrast, on the same caterpillar-conditioned soil, herbivory suppressed *MYB28* 90 min after infestation (47% decrease; p < 0.001 for both soil treatments). This effect disappeared after 150 min. Overall, we observed that caterpillar-induced PSF reduced defence gene expression in the JA and SA pathways with temporal dynamics in the first 2.5 h after infestation.

## Discussion

We investigated the effects of caterpillar-induced changes in the rhizosphere soil on plant defence against insects. Our results showed that *M. brassicae* caterpillars induced changes in the rhizosphere soil that increased resistance in the wild *B. oleracea* accession Durdle Door, which only became apparent after two weeks of caterpillar feeding. In contrast, the cultivated *B. oleracea* accession Rivera became more susceptible to *M. brassicae* on caterpillar-conditioned soil, and this effect was largest at high caterpillar densities in the feedback phase. Susceptibility in this accession seemed to be caused by reduced defence-gene expression in JA biosynthesis and downstream signalling pathways during the onset of plant defence. Moreover, resistance in Rivera plants was not influenced by PSF in different soil types.

We hypothesised that caterpillar-induced PSF responses would enhance resistance against *M. brassicae* in the wild Durdle Door accession compared to the Rivera cultivar. While Durdle Door plants exhibited higher PSF-induced resistance than Rivera plants, we did not expect Rivera to become more susceptible on caterpillar-conditioned soil compared to control soil. Based on our study it is not possible to say whether domestication caused the difference in PSF effect, but in line with our study, intraspecific variation in PSF between cultivated and wild accessions has been found before. For example, in a study with 30 wild and domesticated tomato genotypes and *Manduca sexta* caterpillar herbivory in the feedback phase, domesticated tomato plants suffered more from negative PSF effects on plant growth than wild tomato relatives. No PSF effects were observed on *M. sexta* performance (Carrillo et al. 2019). In another study, 10 different crop species and their wild relatives induced soil legacies that had opposing PSF responses. Domesticated crops had high nematode infection and low mycorrhizal colonisation on domesticated crop-conditioned soil while the opposite pattern was observed for the wild relative on progenitor-conditioned soil (Martin-Robles et al. 2020). Domestication may have resulted in the loss of traits related to PSF, such as the interactions with beneficial microorganisms (Perez-Jaramillo et al. 2016; Porter and Sachs 2020). It remains to be tested with multiple wild and cultivated accessions whether the genotype-specific PFS effects found in our study can be attributed to domestication.

The difference between the two cabbage accessions in mediating caterpillar-induced PSF may be caused by different compositions of the root exudates. Specific metabolites in root exudates are known to be selective, as they can function as anti-microbial to some, while other microorganisms are not affected by these compounds or can even metabolise them. In this way, changes in the root exudates can reshape the soil microbiome composition that can affect the outcome of PSF (Bai et al. 2022; Bouwmeester et al. 2025). Herbivory, for instance, may influence root exudation by activating the JA pathway (Carvalhais et al. 2015, 2017; Rios et al. 2021) that acts also as a key phytohormone in plant defence against chewing herbivores (Erb and Reymond 2019; Howe and Jander 2008). Durdle Door roots are known to contain relatively high concentrations of glucosinolates (GSLs) (Kabouw et al. 2010; Van Geem et al. 2015). Several studies have shown that aboveground herbivory can induce GSL biosynthesis in both the roots and shoots (Hopkins et al. 2009; Karssemeijer et al. 2022b; Soler et al. 2007) and also phloem transport of GSLs has been observed from shoot to root (Andersen et al. 2013). There, GSLs may affect the soil microbiome because these metabolites and their derivatives have anti-microbial properties and are shown to be excreted into the rhizosphere (Chroston et al. 2024; DeWolf et al. 2023; Jacoby et al. 2021; Schreiner et al. 2011; Xu et al. 2017). So far, GSL-mediated changes in the soil microbiome have not been found to affect aboveground resistance to herbivores (DeWolf et al. 2023). Alternatively, the outcome of PSF can be driven by accumulation of metabolites or changes in nutrient states of the soil (Wagner and Mitchell-Olds 2023). This is less likely to have happened in our experiments as the rhizosphere soil was 10 or 100 times diluted with γ-sterile soil before use in the feedback phase. This treatment should have diluted metabolites from the rhizosphere and restored the original nutrient content of the soil. Therefore, we consider it most likely that the microbiome recolonised the soil after inoculation and this, in turn, induced plant resistance in Durdle Door and susceptibility in Rivera to *M. brassicae*. We can only speculate which microorganisms may play a role in plant defence modulation. Inoculation of microorganisms have shown that pathogens, ISR-inducing rhizobacteria and plant-growth-promoting rhizobacteria can induce plant resistance against insects (Biere and Goverse 2016; Cachapa et al. 2021; Friman et al. 2021b; Pangesti et al. 2016). Which microorganisms induce resistance in our study system remains to be investigated with amplicon sequencing, microbial isolations and inoculation.

The results on gene expression showed that the unexpected PSF-induced susceptibility to *M. brassicae* in Rivera plants can be explained by downregulation of the JA-biosynthesis and downstream signalling pathways in the first few hours after induction. These suppressive effects cascaded into the ERF- and MYC-branch and resulted in downregulation of GSL biosynthesis genes. Previous research has shown that the rhizosphere microbiome can indeed induce changes in foliar GSL composition via the JA pathways (DeWolf et al. 2023; Pangesti et al. 2016; Yang et al. 2025). For instance, the JA biosynthesis gene *LOX2* and GSL biosynthesis gene *MYB28* can be downregulated in leaves of *B. oleracea* plants growing on caterpillar- and aphid-conditioned soil, even in the absence of herbivory in the feedback phase (Friman et al. 2021a). In contrast to these studies, we observed similar PSF effects on caterpillar-conditioned soil, but only on plants subjected to herbivory. Rhizobacteria-induced effects on the JA pathway have also been reported in *A. thaliana* after *Pseudomonas simiae* WCS417r (before called *P. fluorescens*) inoculation, but here LOX2 and PDF1.2 were upregulated in response to *M. brassicae* feeding (Pangesti et al. 2015). In addition to JA, microorganisms could also activate other pathways, such as the SA pathway, which may suppress the JA pathway (Eichmann et al. 2021; Thaler et al. 2012). However, it is unlikely that SA supressed the JA pathway in our experiment as SA biosynthesis and downstream signalling were not upregulated by soil conditioning or herbivory. Transcriptome and metabolome studies could shed more light on the interactions of pathways and why the JA pathway is downregulated by caterpillar-conditioned soil in the cultivated accession Rivera. We did not analyse gene expression in the wild Durdle Door accession. Based on gene expression analysis in Rivera, we speculate that the PSF-induced resistance may be induced by upregulation of JA biosynthesis and downstream signalling. This could lead to higher GSL production and lower caterpillar performance. However, the underlying mechanisms in wild and cultivated accessions may not be directly comparable as selection for traits important for plant breeding are not the same as selection forces experienced under natural conditions. This could have resulted in the loss of defence traits in cultivated accessions (Bernal and Medina 2018; Whitehead et al. 2017). Future research should analyse gene expression in wild accessions such as Durdle Door to understand the mechanisms driving PSF-induced resistance against caterpillars. In Rivera we analysed gene expression in the first 1.5 h after caterpillar induction. This is an important phase in plant defence signalling during which herbivores can be affected by earlier or higher defence gene activation due to priming or legacies that are induced by microorganisms or previous herbivory (Hickman et al. 2017; Karssemeijer et al. 2022a; Pangesti et al. 2015). This initiation of defence gene activation can have long-lasting consequences for plant resistance and herbivore performance (Kumari et al. 2019). Future research should investigate how PSF-affected defence gene expression develops after the first 2.5 h of caterpillar induction.

Another surprising result was that *M. brassicae* feeding on control soil did not upregulate the JA pathway as would be expected from chewing herbivory (Erb and Reymond 2019; Howe and Jander 2008). Several herbivorous insect species can supress plant defences by releasing effectors produced by themselves or their endosymbionts (Li et al. 2024). For instance, *Helicoverpa armigera* caterpillars have a venom-like protein (HARP1) in their oral secretion that blocks JA signalling (Chen et al. 2019). It remains to be tested whether our *M. brassicae* line has acquired such a strategy, and whether this strategy already affects plant defence in first 2.5 h after caterpillar induction.

We expected that the effect of caterpillar-induced PSF on plant resistance against *M. brassicae* would be larger at higher damage intensities in the feedback phase, i.e. more caterpillars or longer infestation time. Indeed, caterpillar-induced PSF only affected resistance to *M. brassicae* in Rivera plants at high caterpillar density in the first two weeks of *M. brassicae* development. A similar effect has been found before, where the amount of damage on receiver plants affected the magnitude of PSF on these plants (Heinze et al. 2019). Furthermore, in line with our hypothesis, PSF-induced resistance to caterpillars in Durdle Door plants became more pronounced when caterpillar damage increased over time. Plant-soil feedback effects are not necessarily stable, but are expected to change over time as the underlying soil-borne mechanisms develop with time (Hannula et al. 2021; Kardol et al. 2013), and the microbiome may need time to develop its insect-suppressive capacity. Therefore, it may take some time for the PSF effects to become visible although fast responses are observed as well (Hu et al. 2025). Slower responses may particularly be observed when PSF effects are subtle, as was the case in this study. This subtle effect may have occurred because caterpillars were feeding less on Durdle Door compared to Rivera and thereby developed slower. In addition, increased damage due to more caterpillars or longer infestation time may stimulate plants to invest extra resources in the interaction with a beneficial soil microbiome (Schultz et al. 2013; Xing et al. 2024), which can result in increased plant resistance (Pieterse et al. 2014). In addition, increased investment in the soil microbiome may result from changed source-sink relationships. There is the risk that if more carbon is transported from leaves to roots to mobilize the soil microbiome, this reallocation of carbon may limit upregulation of plant defence in leaves due to resource depletion, unless plant can increase photosynthesis rate in non-damaged leaves (Schultz et al. 2013). Plant-soil feedback effects upon caterpillar infestation may also need time to develop in both natural and agricultural systems. We hypothesise that PSF-induced resistance may depend on severity and timing of the infestation and can persist throughout the season thereby also affecting the plant that induces the PSF. *Brassica oleracea* plants have a long growing season and can potentially be affected by a delayed soil legacy that affects plant resistance. Future research should investigate this hypothesis.

Overall, we showed that a wild cabbage plant can increase resistance to a caterpillar pest by conditioning the soil while resistance was reduced in a cultivated accession, most likely by downregulating the JA biosynthesis and signalling pathways. The underlying mechanisms of PSF and when they convey positive or negative effects on the plant should be further investigated as they can be a key element in enlarging rhizosphere-induced resistance in crop plants against insect pests, for instance via microbial inoculum (Nerva et al. 2022). In addition, the genetic basis in plants of PSF-enhanced resistance should be revealed to breed insect-resistant crops. In these ways, PSF strategies could be employed to reduce insect pests and offer sustainable alternatives for the use of insecticides (Bakker et al. 2012; Nerva et al. 2022).

## Supplementary Information

Below is the link to the electronic supplementary material.

Supplementary file1 (DOCX 469 KB)

## Data Availability

Collected data for this manuscript is available upon reasonable request.

## Abbreviations

PSF: Plant-soil feedback
CP: Conditioning phase
FP: Feedback phase
JA: Jasmonic acid
SA: Salicylic acid
GSL: Glucosinolate
RT-qPCR: Real-time quantitative PCR
Exp: Experiment
RGE: Relative gene expression
