# Supplementary material for: Caterpillar-induced plant-soil feedback affects resistance in wild and cultivated cabbage
Source: Plant Soil. 2026 Feb 11;520(2):1721–40. doi: 10.1007/s11104-026-08355-4 (PMC13065549; doi:10.1007/s11104-026-08355-4)
Supplement: Supplementary file 1 — Supplementary file1 (DOCX 469 KB) [file 11104_2026_8355_MOESM1_ESM.docx]

**Supplementary Information**

Article Title: Caterpillar-induced plant-soil feedback affects resistance in wild and cultivated cabbage

Kris A. de Kreek, Rieta Gols, Johannah M. de Zeeuw, Ilva van Dam, Rob Nijhof, Brigitte S. Noordijk, Marcel Dicke, and Karen J. Kloth*

Laboratory of Entomology, Wageningen University & Research, PO Box 16, 6700 AA, Wageningen, the Netherlands

*Corresponding author: karen.kloth@wur.nl (Karen J. Kloth)

ORCID

KA de Kreek: 0009-0000-7158-7775

R Gols: 0000-0002-6839-8225

M Dicke: 0000-0001-8565-8896

KJ Kloth:: 0000-0002-0379-5473

**Table S1** Soil bioavailable chemical composition of old batch of “MiCRop” soil (stored for six months as described above), new batch of “MiCRop” soil (freshly collected just before the experiment and processed as described above), and “Reijerscamp” soil

| **Soil** | **Al** | **Cu** | **Fe** | **K** | **Mg** | **Mn** | **Na** | **P** | **S** | **Zn** | **N-NH4** | **N-(NO3+NO2)** | **Nts** | **P-PO4** |
| --- | --- | --- | --- | --- | --- | --- | --- | --- | --- | --- | --- | --- | --- | --- |
|  | [mg/kg] | [mg/kg] | [mg/kg] | [mg/kg] | [mg/kg] | [mg/kg] | [mg/kg] | [mg/kg] | [mg/kg] | [mg/kg] | [mg/kg] | [mg/kg] | [mg/kg] | [mg/kg] |
| *Minimum detection limit* | *0.5* | *-* | *3* | *3* | *0.5* | *0.1* | *3* | *1* | *0.6* | *0.3* | *1* | *0.5* | *4* | *0.4* |
| “MiCRop” old | 2.3 | 0.04 | 0 | 52 | 52.2 | 2.71 | 8 | 1.3 | 3.1 | 0.6 | 0.5 | 13.1 | 18 | 0.8 |
| “MiCRop” fresh | 0.4 | 0.02 | 0 | 65 | 56.0 | 0.37 | 3 | 1.1 | 1.0 | 0.0 | 2.1 | 2.3 | 9 | 0.8 |
| “Reijerscamp” | 1.8 | 0.01 | 0 | 79 | 103 | 1.82 | 10 | 2.2 | 2.2 | 0.9 | 3.4 | 7.2 | 21 | 1.3 |

**Table S2** Grain size of old batch of “MiCRop” soil (stored for six months as described above), new batch of “MiCRop” soil (freshly collected just before the experiment and processed as described above), and “Reijerscamp” soil determined by laser defraction. Sand has a grain size < 50μm, silt 2-50μm and clay < 2μm

| **Soil** | **Sand (%)** | **Silt (%)** | **Clay (%)** |
| --- | --- | --- | --- |
| “MiCRop” old | 80.53 | 17.60 | 1.87 |
| “MiCRop” fresh | 78.99 | 18.74 | 2.27 |
| “Reijerscamp” | 63.19 | 30.51 | 6.30 |

**Table S3** Detailed information on each experiment (CP: conditioning phase; FP: feedback phase, DRST: dry rhizosphere soil transfer)

| **Experiment** | **Start date experiment** | **Phase** | **Accession** | **Number of plants per treatment** | **Soil conditioning method** | **Time before infestation (days)** | **Number of caterpillars per plant** | **Infestation time (days)** |
| --- | --- | --- | --- | --- | --- | --- | --- | --- |
| Host plant resistance | Mar 2022 | No PSF | Durdle Door | 6 |  | 26 | 10 | 5 |
|  |  |  | Rivera | 6 |  | 26 | 10 | 5 |
| 1. Density effect | Jul 2022 | CP | Durdle Door | 60 |  | 23 | 16 | 8 |
|  |  |  | Rivera | 60 |  | 23 | 8 | 8 |
|  |  | FP | Durdle Door | 12 | Soil slurry | 25 | 4 or 8 | 10 |
|  |  |  | Rivera | 11 | Soil slurry | 22 | 1, 2 or 4 | 10 |
| 2. Effect of infestation time | Jan 2023 | FP | Durdle Door | 30 | Bulk soil | 35 | 1^st^ week: 2; later: 1 | 24 |
|  | Sep 2023 | CP | Durdle Door | 60 |  | 28 | 4 | 14 |
|  |  |  | Rivera | 60 |  | 28 | 3 | 14 |
|  |  | FP | Durdle Door | 30 | DRST | 41 | 1 | 25 |
|  |  |  | Rivera | 30 | DRST | 41 | 1 | 25 |
| 3. Different soil types | Sep 2023 | CP | Rivera | 36 |  | 29 | 3 | 13 |
|  |  | FP | Rivera | 20 | DRST | 73 | 1 | 19 |
| 4. Gene expression analysis | Sep 2023 | CP | Rivera | 155 |  | 29 | 3 | 14 |
|  |  | FP | Rivera | 30 | DRST | 35 | 10 | 60, 90, 150 min |

**Table S4** Information on RT-qPCR primers

| **Gene acronym** | **Gene amplified** | **Forward sequence (5’ – 3’)** | **Reverse sequence (5’ – 3’)** | **Efficiency (%)** |
| --- | --- | --- | --- | --- |
| *AOS* | Bo2g116210 | ACC GCT TGC GAC TAG GGA TC | CAA AGT CCT TAC CGG CGC AC | 116.8 |
| *MYC2* | Bo5g086990 | GGC TGG ACC TAC GCT ATA TTC TGG | AGA AAA ACC ACT CCG TAT CCG T | 114.0 |
| *PDF1.2* | Bo2g086460 | CCC TTC TCT TCG CTG CTC TT | ACT CCT GAC CAT GTC CCA CT | 141.2 |
| *CYP81F4* | NA | GTT TTG GAG GTA GGG CAG GT | GCA TCA TGT TTG CGG CAT CA | 100.6 |
| *MYB28* | Bo2g161590 | CGG GAG AGA TGA GCA CAA TAC G | CAG CCC TCG AAG TTT CCT ATC A | 132.3 |
| *ICS1* | XM_013732875.1 | TTC TGT TGT GGT GAG GTT ACG | TCT CGA CAG CAG GGT CAT AC | 105.1 |
| *BGL2* | NA | GGT CGG TAA CGA GGT GAA AC | GTG GTG GCT CCC ATG TCT AT | 103.5 |
| *SAR1a* | Bo3g052780 | ATC TCT AGC CAC CGT TCC CT | TTC CTG ACG ATG CTG CAC AT | 97 |
| *PER4* | Bo7g095750 | TAT CCT CTG CAG CCT CCT CA | ACA CAC AGA CTG AAG CGT CC | 94 |

**Table S5** Statistical tests per experiment (DD: Durdle Door; RI: Rivera; RGE: relative gene expression; FP: feedback phase)

| **Experiment** | **Factors** | **Dependent variable** | **Test** | **Data transformation** | **Note** |
| --- | --- | --- | --- | --- | --- |
| Host plant resistance | accession | fraction surviving caterpillars, leaf damage, total leaf area and root biomass | two-sample t-test |  |  |
|  |  | caterpillar weight | GLMM gamma distribution | ln |  |
| 1. Density effect | soil treatment and caterpillar number | caterpillar weight | LMM | ln (only DD) | DD and RI analysed separately |
|  |  | leaf damage | LMM | sqrt (only DD) |  |
|  |  | fraction surviving caterpillars | GLMM beta binomial distribution (DD), GLM binomial distribution (RI) |  |  |
|  |  | total leaf area | LMM (DD), LM (RI) |  |  |
|  |  | root biomass | GLMM gamma distribution (DD), LMM (RI) |  |  |
| 2. Effect of infestation time | soil treatment | exponential growth rate | two-sample t-test |  | experiment only with DD |
|  |  | caterpillar weight at 21 dpi | Mann Whitney-U test |  |  |
| 2. Effect of infestation time | time (only DD) and soil treatment | caterpillar weight | LMM (DD), GLMM gamma distribution (RI) | ln | DD and RI analysed separately |
| 3. Different soil types | soil type and soil treatment | linear growth rate | GLMM gamma distribution |  |  |
| 4. Gene expression analysis | time after caterpillar introduction, soil treatment, caterpillar infestation in FP | RGE *AOS* | GLMM gamma distribution | sqrt | each gene was analysed separately |
|  |  | RGE *MYC2, PDF1.2*, *MYB28* | LMM | ln |  |
|  |  | RGE *CYP81F4*^a^, *ICS1*, *BGL2* | GLMM gamma distribution | ln |  |

^a^ Slight unequal variance occurred when fitting the full model. New models were run for each time point separately, and these models were fitting the data well. Nevertheless, the model outcomes were very similar to the initial model, so we decided to stick to the full model for consistency

**Table S6**. Statistical outcomes of Wald chi square tests on (G)LMM models of RT-qPCR data, including χ^2^, degrees of freedom (df) and p values of relative gene expression of *AOS*, *MYC2*, *PDF1.2*, *CYP81F1*, *MYB28*, *ICS1* and *BGL2* genes in Rivera at 60, 90 and 150 minutes after *M. brassicae* introduction (Time) in feedback phase of a the PSF experiment. Plants were growing on caterpillar-conditioned or control-conditioned soil (Soil) and with or without caterpillar infestation (Caterpillar or Cat). Biological replicates: n= 10 per treatment combination.

|  |  | ***AOS*** |  | ***MYC2*** |  | ***PDF1.2*** |  | ***CYP81F4*** |  | ***MYB28*** |  | ***ICS1*** |  | ***BGL2*** |  |
| --- | --- | --- | --- | --- | --- | --- | --- | --- | --- | --- | --- | --- | --- | --- | --- |
| **Factor** | **df** | **χ^2^** | **p value** | **χ^2^** | **p value** | **χ^2^** | **p value** | **χ^2^** | **p value** | **χ^2^** | **p value** | **χ^2^** | **p value** | **χ^2^** | **p value** |
| Time | 2 | 2.008 | 0.366 | 1.298 | 0.522 | 15.906 | **< 0.001** | 2.950 | 0.229 | 22.744 | **< 0.001** | 32.086 | **< 0.001** | 18.424 | **< 0.001** |
| Soil | 1 | 14.028 | **< 0.001** | 12.804 | **< 0.001** | 6.670 | **0.010** | 8.161 | **0.004** | 1.631 | 0.201 | 10.747 | **0.001** | 3.210 | 0.073 |
| Caterpillar | 1 | 4.423 | **0.035** | 6.535 | **0.011** | 0.097 | 0.755 | 5.456 | **0.019** | 23.796 | **< 0.001** | 0.934 | 0.334 | 4.165 | **0.041** |
| Time:Soil | 2 | 14.180 | **0.001** | 1.537 | 0.464 | 0.572 | 0.751 | 0.806 | 0.668 | 11.999 | **0.002** | 1.770 | 0.413 | 0.607 | 0.738 |
| Time:Cat | 2 | 0.238 | 0.888 | 7.484 | **0.024** | 23.270 | **< 0.001** | 6.413 | **0.040** | 31.312 | **< 0.001** | 6.493 | **0.039** | 6.866 | **0.032** |
| Soil:Cat | 1 | 1.201 | 0.273 | 18.213 | **< 0.001** | 5.052 | **0.025** | 12.970 | **< 0.001** | 2.943 | 0.086 | 0.324 | 0.569 | 5.579 | **0.018** |
| Time:Soil:Cat | 2 | 4.003 | 0.135 | 8.740 | **0.013** | 0.493 | 0.782 | 0.444 | 0.801 | 0.024 | 0.988 | 20.343 | **< 0.001** | 0.773 | 0.679 |

**
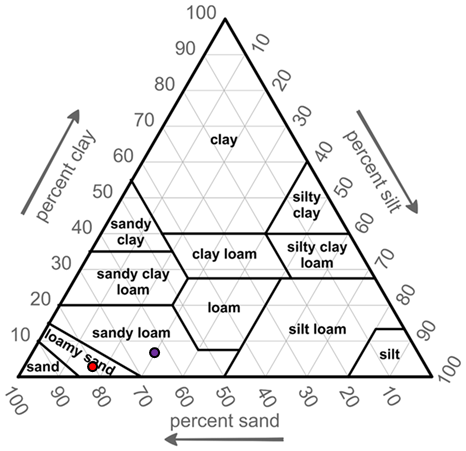
**

**Fig. S1** USDA soil texture triangle with soil texture of MiCRop (red dot; average of old and fresh) and Reijerscamp soil (purple dot) indicated. Grain size was determined by laser defraction (Figure adapted from (roenendyk et al. 2015)


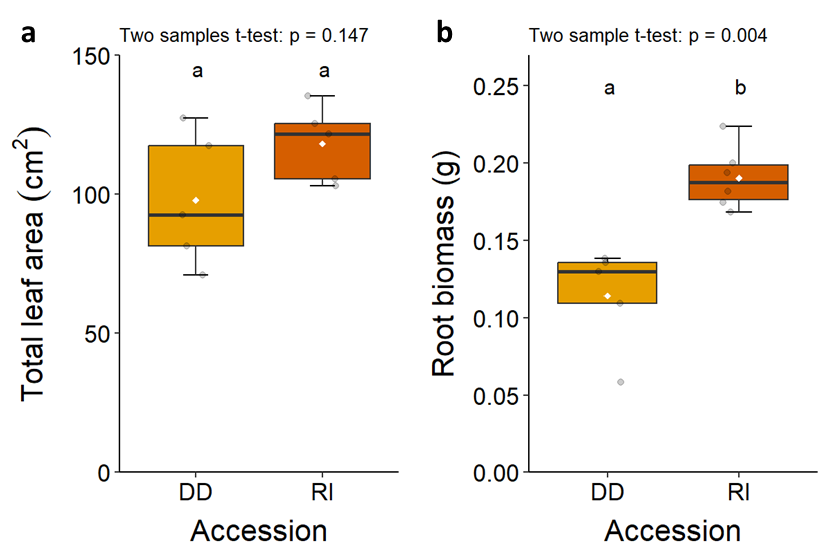


**Fig. S2** A comparison of total leaf area (**a**; DD and RI: n = 5) and root biomass (**b**; DD: n = 5, RI: n = 6) between Durdle Door (DD) and Rivera (RI). Plants were infested by *Mamestra brassicae* caterpillars. Boxes of boxplots represent the second and third quartile of the data, whiskers represent at maximum 1.5 times the distance between the first and third quartiles, grey dots display single data points, and white diamonds indicate mean per treatment. Letters above boxplots present statistical differences between treatments based on a Tukey post-hoc test (α = 0.05)



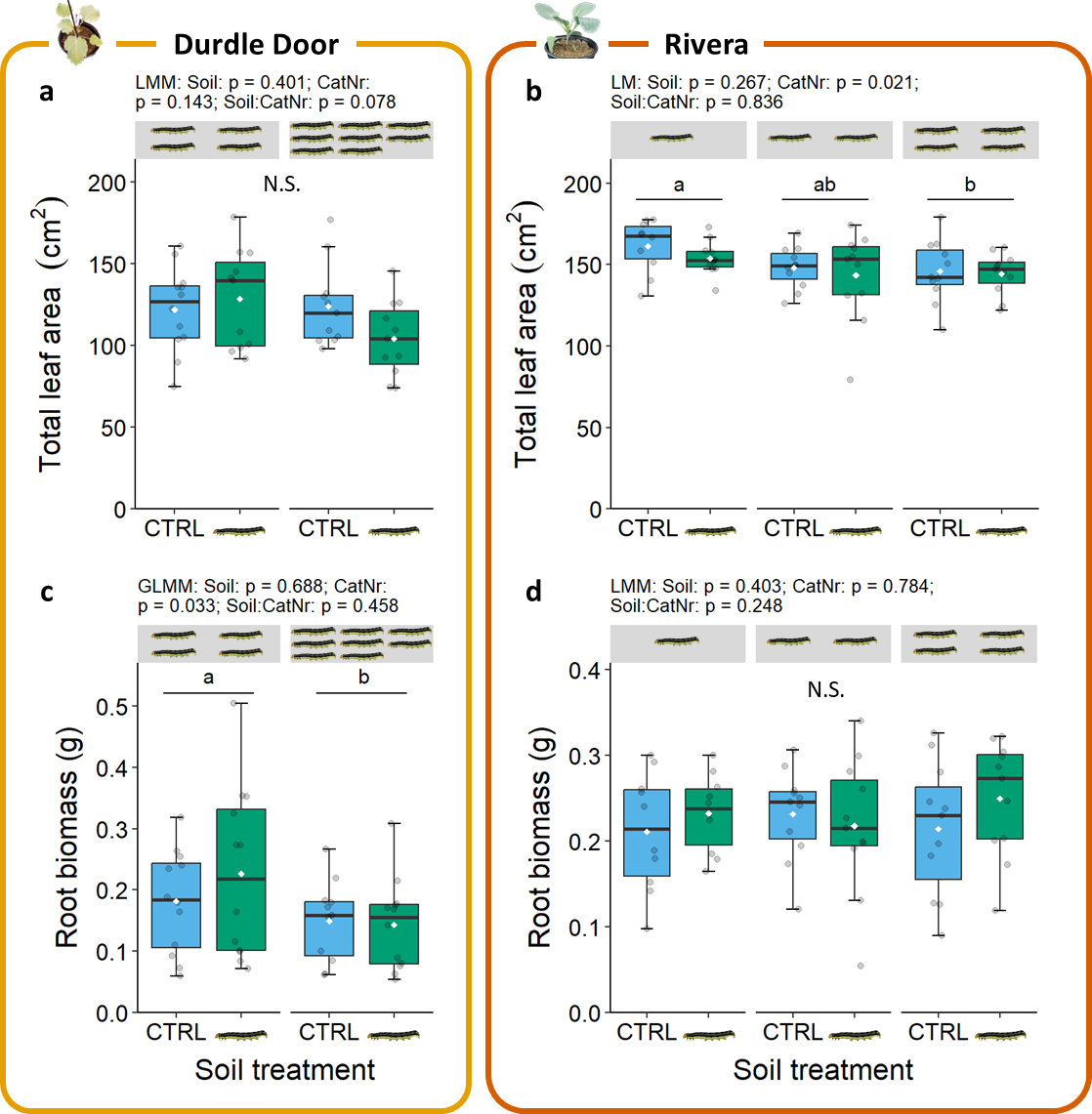


**Fig. S3** Effects of soil legacy in control-conditioned (CTRL) or caterpillar*-*conditioned (
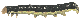
) soil on total leaf area (**ab**) and root biomass (**cd**) of Durdle Door (left panels) and Rivera (right panels) plants at different *M. brassicae* densities (CatNr) in feedback phase (Experiment 1). Caterpillars were allowed to feed for 10 days. Densities are indicated by the number of caterpillars above the boxplots (Durdle Door: 4 and 8 caterpillars; Rivera: 1, 2 and 4 caterpillars). Durdle Door: n = 11-12; Rivera: n = 10-11. Boxes of boxplots represent the second and third quartile of the data, whiskers represent at maximum 1.5 times the distance between the first and third quartiles, grey dots display single data points, and white diamonds indicate mean per treatment. Letters above boxplots present statistical differences between treatments based on a Tukey post-hoc test (α = 0.05), N.S. means no statistical differences

References

Groenendyk DG, Ferre TP, Thorp KR, Rice AK (2015) Hydrologic-process-based soil texture classifications for improved visualization of landscape function. PLoS One 10: e0131299 https://doi.org/10.1371/journal.pone.0131299.
